# Ceramides and pro-inflammatory cytokines for the prediction of acute coronary syndrome: a multi-marker approach

**DOI:** 10.1186/s12872-023-03690-1

**Published:** 2024-01-13

**Authors:** Huiqing Liang, Fangjiang Li, Liang Zhang, Lin Li, Bingyan Guo

**Affiliations:** 1 Department of Internal Medicine, Hebei Medical University, Shijiazhuang, 050000 China; 2 Department of Cardiology, The First Affiliated Hospital of Hebei North University, Zhangjiakou, 075000 China; 3 Department of Cardiology, Beijing Anzhen Hospital, Capital Medical University, Beijing, 100020 China; 4 Beijing Health Biotech Co. Ltd, Beijing, 102200 China; 5 Department of Internal Medicine, Hebei Medical University, No 361 Zhongshan East Road, Changan District, Shijiazhuang, 050000 China; 6 Department of Cardiovascular Medicine, The Second Hospital of Hebei Medical University, Heping West Road No. 215, Shijiazhuang, 050000 China

**Keywords:** Ceramides, Pro-inflammatory cytokines, Acute coronary syndrome

## Abstract

**Background:**

There is a growing body of evidence supporting the significant involvement of both ceramides and pro-inflammatory cytokines in the occurrence and progression of acute coronary syndrome (ACS).

**Methods:**

This study encompassed 216 participants whose laboratory variables were analysed using standardised procedures. Parameters included baseline serum lipid markers, comprising total cholesterol, low-density lipoprotein-cholesterol, high-density lipoprotein-cholesterol, triglycerides (TGs), lipoprotein(a) (LPa), fasting blood glucose, B-natriuretic peptide and hypersensitive C-reactive protein. Liquid chromatography-tandem mass spectrometry measured the concentrations of plasma ceramides. Enzyme-linked immunosorbent assay quantified tumour necrosis factor-α (TNF-α), interleukin 6 (IL6) and IL8. The correlation between ceramides and inflammatory factors was determined through Pearson’s correlation coefficient. Receiver operating characteristic (ROC) curve analysis and multivariate logistic regression evaluated the diagnostic potential of models incorporating traditional risk factors, ceramides and pro-inflammatory cytokines in ACS detection.

**Results:**

Among the 216 participants, 138 (63.89%) were diagnosed with ACS. Univariate logistic regression analysis identified significant independent predictors of ACS, including age, gender, history of diabetes, smoking history, TGs, TNF-α, IL-6, ceramide (d18:1/16:0), ceramide (d18:1/18:0), ceramide (d18:1/24:0), ceramide (d18:1/20:0) and ceramide (d18:1/22:0). Multivariate logistic regression analysis revealed significant associations between gender, diabetes mellitus history, smoking history, LPa, IL-6, ceramide (d18:1/16:0) and ACS. Receiver operating characteristic analysis indicated that model 4, which integrated traditional risk factors, IL-6 and ceramide (d18:1/16:0), achieved the highest area under the curve (AUC) of 0.827 (95% CI 0.770–0.884), compared with model 3 (traditional risk factors and ceramide [d18:1/16:0]) with an AUC of 0.782 (95% CI 0.720–0.845) and model 2 (traditional risk factors and IL-6), with an AUC of 0.785 (95% CI 0.723–0.846) in ACS detection.

**Conclusions:**

In summary, incorporating the simultaneous measurement of traditional risk factors, pro-inflammatory cytokine IL-6 and ceramide (d18:1/16:0) can improve the diagnostic accuracy of ACS.

## Introduction

Acute coronary syndrome (ACS) encompasses various types of myocardial ischaemia, including ST-segment elevation myocardial infarction (STEMI), non-STEMI and unstable angina pectoris (UAP). The condition is primarily caused by the destabilisation of coronary atherosclerotic plaques [1, 2]. Emerging evidence suggests that an upregulated inflammatory response and abnormal metabolism of specific lipid molecules play significant roles in the formation, rupture and subsequent development of ACS. In addition to established traditional lipid biomarkers, such as low-density lipoprotein (LDL-C), high-density lipoprotein (HDL-C) and triglycerides (TGs), several metabolomic and lipidemic indicators, including ceramides and apolipoproteins, have been implicated in the occurrence and progression of ACS [3–5].

Ceramide, a sphingolipid derivative of sphingomyelinase, assumes a critical function in preserving the structural integrity of cells and acts as a bioactive lipid participating in diverse cellular signalling pathways related to cell proliferation, differentiation and apoptosis. Substantial research has established a link between ceramides and multiple atherosclerotic processes, encompassing lipoprotein aggregation, cholesterol accumulation in macrophages, the modulation of nitric oxide synthesis, the generation of superoxide anions and the regulation of cytokine expression [6–9]. Notably, ceramides have been implicated in acute coronary events by fostering the infiltration of oxidised LDL-C into vascular walls, monocyte adhesion, atherosclerotic plaque formation and the expansion of the lipid-rich core, rendering it more susceptible to rupture [10]. Intracoronary imaging studies have predominantly identified ceramides within the thin fibrous cap of atheromatous plaques with necrotic cores [11, 12]. Plasma ceramides have exhibited a positive and independent correlation with plaque rupture and erosion in patients suffering from acute myocardial infarction (AMI), as evaluated by optical coherence tomography [13]. Moreover, specific molecular lipid species, particularly ceramide (d18:1/16:0), have been linked to the fraction of necrotic core tissue and lipid core burden in coronary atherosclerosis, serving as predictive markers for the 1-year clinical outcome following coronary angiography (CAG) [5]. Bolstered by both theoretical evidence and empirical findings, ceramide (d18:1/16:0), ceramide (d18:1/18:0) and ceramide (d18:1/24:1) and their ratios to ceramide (d18:1/24:0) have emerged as novel risk indicators in patients diagnosed with confirmed coronary heart disease [14].

In addition, the progression of atherosclerotic cardiovascular disease is influenced by systemic vascular inflammation, which contributes to multiple maladaptive processes [15]. Interleukin-6 (IL-6), a pro-inflammatory cytokine, has been proposed as a potential predictor of coronary artery disease (CAD) severity and has been associated with plaque burden, as assessed by intracoronary imaging [16, 17]. Furthermore, evidence suggests that the activation of the inflammatory pathway is crucial for ceramide biosynthesis [18]. In-vitro experiments have also demonstrated the interaction between ceramides and cytokines in various cellular pathways, highlighting their involvement in inflammation [19–21]. Given the interplay between pro-inflammatory cytokines and ceramides, along with their significant roles in the occurrence and progression of ACS, it is conceivable that there is a substantial overlap in the biological processes triggered by these factors in patients diagnosed with ACS.

In summary, we speculated that the concurrent measurement of traditional risk factors, pro-inflammatory cytokines such as tumour necrosis factor-alpha (TNF-α) and IL-6, along with ceramides, could improve the diagnostic accuracy of ACS. However, limited research has explored the relationship between inflammatory factors and ceramides in patients with ACS, as well as the potential diagnostic benefits of concurrently measuring pro-inflammatory cytokines, such as TNF-α and IL-6, alongside ceramides. Therefore, the objective of our study was to investigate the correlation between ceramides and pro-inflammatory cytokines in patients with ACS and to evaluate the potential added value of combining ceramide and pro-inflammatory cytokine testing for early ACS diagnosis.

## Method and materials

### Study design and participants

**Fig. 1:**
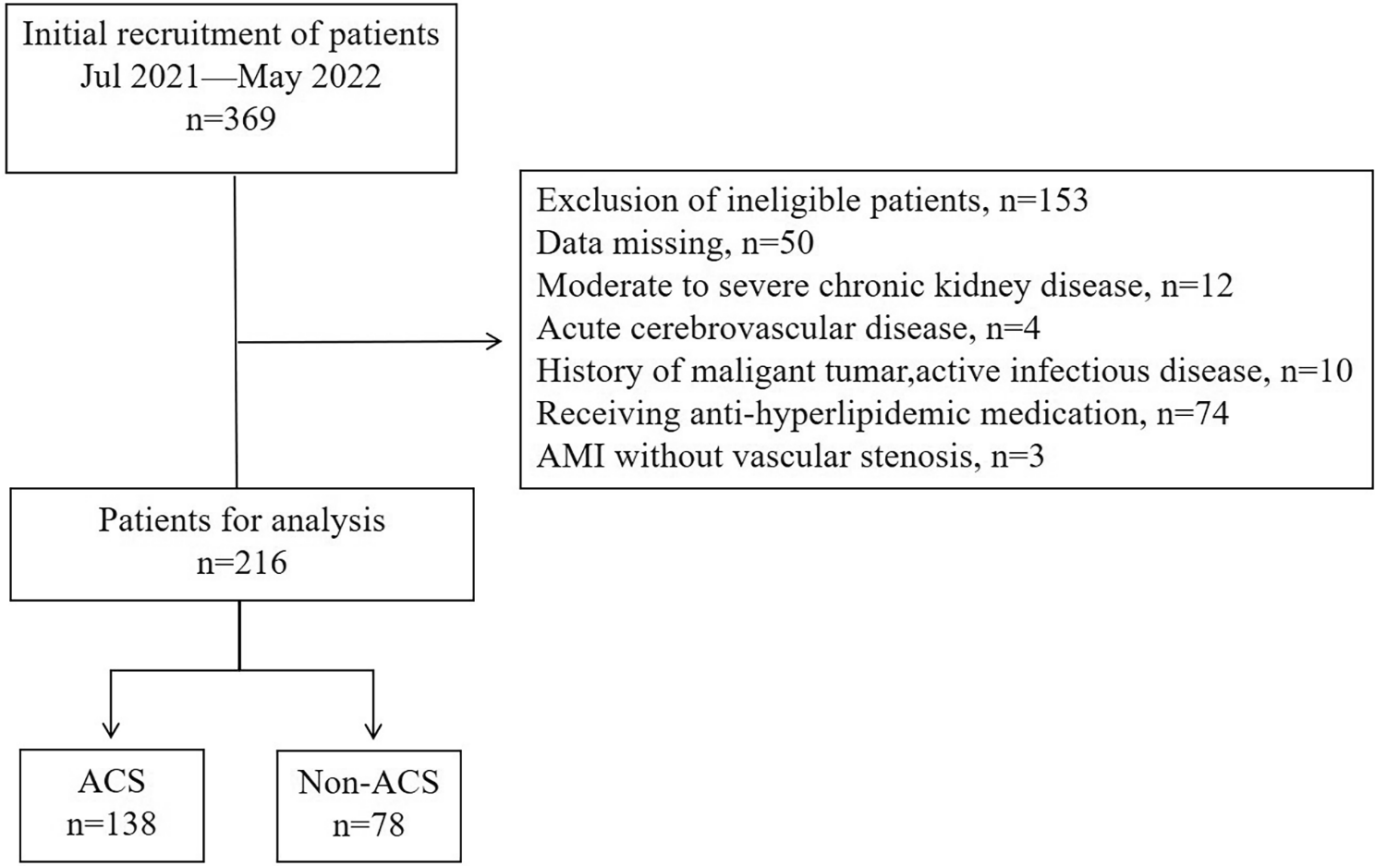
Flow chart. ACS, acute coronary syndrome; AMI,acute myocardial infarction

This observational study involved the enrolment of 216 patients who were admitted and underwent CAG for suspected CAD between July 2021 and May 2022 at the Second Hospital of Hebei Medical University, China. The exclusion criteria encompassed moderate to severe chronic kidney disease (estimated glomerular filtration rate ≤ 60 mL/min/1.73 m²), chronic heart failure, acute cerebrovascular disease, a history of malignant tumours and active infectious diseases. Patients who had been prescribed anti-hyperlipidaemic medication or met the diagnostic criteria for AMI without evidence of vascular stenosis were also excluded from the study. For a comprehensive overview of the detailed inclusion and exclusion processes, please refer to Fig. 1.

The inclusion criterion was patients suspected of having CAD by clinical doctors based on their clinical symptoms (e.g. chest pain, dyspnoea).

Two highly skilled cardiologists independently reviewed all coronary angiograms, and a diagnosis of ACS was determined based on a combination of clinical symptoms, electrocardiographic changes, cardiac biomarkers and findings from CAG that indicated the presence of significant stenosis (≥ 50%) in one or more coronary arteries, following the recommended criteria outlined in the European Society of Cardiology guidelines [22, 23]. Prior to participation in the study, written informed consent was obtained from all of the enrolled participants. The study protocol strictly adhered to the principles outlined in the Declaration of Helsinki and received approval from the Ethics Committee of the Second Hospital of Hebei Medical University (ethics approval number: W2021041).

### Preparation of blood samples

After a minimum fasting period of 12 h following admission, venous blood samples were collected from the patients. The blood was drawn from peripheral veins using ethylenediaminetetraacetic acid-containing tubes to preserve the integrity of the samples for the measurement of ceramides and cytokines. To ensure optimal preservation, aliquots of plasma were promptly prepared and stored at a temperature of − 80℃ until the time of analysis.

### Demographic, clinical and laboratory assessments

The study assessed various demographic and traditional risk factors. Demographic variables included age and gender. The traditional risk factors that were evaluated were body mass index (kg/m2), smoking history, medical history of alcohol consumption, diabetes mellitus (DM) and hypertension. In addition, laboratory variables were measured, including baseline serum lipid markers, such as total cholesterol, LDL-C, HDL-C and TGs. Other variables measured were lipoprotein(a) (LPa), hypersensitive C-reactive protein (hs-CRP), fasting blood glucose and B-natriuretic peptide.

### Ceramide measurements

The quantification of ceramides was conducted by a senior laboratory examiner who was blinded to the clinical details of the participants. To extract plasma ceramides, a liquid–liquid extraction method using methanol was employed. The levels of plasma ceramide (d18:1/14:0), ceramide (d18:1/16:0), ceramide (d18:1/18:0), ceramide (d18:1/20:0), ceramide (d18:1/22:0), ceramide (d18:1/24:0) and ceramide (d18:1/24:1) were measured using the Shimadzu LC-20 A system. The Phenomenex Kinetex C18 analytical column (2.6 μm, 3.0 × 50 mm id.) was used coupled with an AB SCIEX triple Quad 4500 tandem mass spectrometer (Applied Biosystems Inc., USA) equipped with an electrospray ion source. The gradient reverse phase chromatography involved mobile phases including liquid chromatography–mass spectrometry (LC–MS) grade water (A) with 0.1% formic acid-mM ammonium acetate water and (B) 0.1% formic acid-2mM ammonium acetate-acetonitrile-isopropyl alcohol (7:3/v:v). Ceramide (d18:1/17:0) was used as the internal standard. The calibration linearity of ceramides was established by plotting the ratio of the peak response of ceramides to the peak response of their respective stable isotope internal standard in working standard solutions against the quantity of ceramides. To ensure the stability of analysis and calibration verification, three levels of standard solutions (QCL, QCM and QCH) were employed as quality controls and were checked every 10 injections. All ceramides exhibited excellent linearity within the calibration range, with correlation coefficients (R^2^) of > 0.99. No matrix interference or carryover was observed during the analysis.

### Cytokine measurements

The plasma levels of TNF-α, IL-6 and IL-8 were quantified using enzyme-linked immunosorbent assay (ELISA) kits obtained from Biotech Pack Analytical in Beijing, China. The ELISA kits had a minimum detectable concentration of < 1.0 pg/mL. After the samples were thawed, the ELISA measurements were conducted by a skilled laboratory examiner. The method employed yielded both intra-assay and inter-assay coefficients of variation of < 15% each, ensuring reliable and consistent results.

### Statistical analyses

Statistical analyses were conducted using the IBM SPSS Statistics version 25.0 software (IBM Corp., Armonk, NY, USA). Continuous variables were presented as mean ± standard deviation, while categorical variables were expressed as percentages. The normality of data was assessed using the Kolmogorov–Smirnov test. For normally distributed continuous variables, the student’s t-test was used for inter-group comparisons, whereas the Mann–Whitney U test was employed for non-normally distributed data. Categorical variables were compared using either the chi-square test or Fisher’s exact test, depending on the sample size of patients in the analysis group. The association between ceramides and pro-inflammatory cytokines was evaluated using Pearson’s correlation coefficient. Receiver operating characteristic (ROC) curves and multivariate logistic regression were used to analyse the clinical accuracy of ceramides combined with cytokines in predicting ACS. The performance and discrimination ability of the four diagnostic models were assessed using the R statistical software version 3.4.3. Statistical significance was considered when the two-sided *P*-value was < 0.05.

## Results

### Demographic, clinical and laboratory findings

Table 1 presents the demographic, clinical and laboratory characteristics of all participants included in the study. Out of the total of 216 participants, 138 were diagnosed with ACS, while the remaining participants were classified as non-ACS. Within the ACS group, 11 individuals (7.97%) had STEMI, 25 (18.12%) had NSTEMI and 102 (73.91%) were diagnosed with UAP. All patients with UAP exhibited major vessel stenosis of > 50% as observed via CAG. In comparison with the non-ACS group, a higher percentage of patients with ACS were men (69.6%), current smokers (44.2%) and had a history of DM (37.0%). Additionally, the ACS group demonstrated significantly higher TG and LPa levels, along with lower levels of HDL-C, in comparison with those without ACS. Furthermore, BNP levels were found to be significantly elevated in patients with ACS (*P* < 0.05).

**Table 1:** Demographic, clinical and laboratory findings of all the included patients

Clinical characteristics	total(n = 216)	ACS (n = 138)	non ACS (n = 78)	*P*
DM(n,%)	66 (30.6)	51 (37.0)	15 (19.2)	0.007
Age	60.61	59.88	61.90	0.170
Gender	131 (60.6)	96 (69.6)	35 (44.9)	0.000
Hypertention(n,%)	130 (60.2)	85 (61.2)	45 (57.7)	0.574
Current smoker(n,%)	74 (34.3)	61 (44.2)	13 (16.7)	0.000
Alcohol status(n,%)	40 (18.5)	31 (22.5)	9 (11.5)	0.047
Presentation of ACS				
STEMI(n,%)	11 (5.09)	11 (7.97)		
NSTEMI(n,%)	25 (11.57)	25 (18.12)		
UAP(n,%)	102 (47.22)	102 (73.91)		
BMI(kg/cm^2^)	26.59 ± 4.27	26.08 ± 3.80	27.51 ± 4.89	0.018
TG(mmol/L)	1.74 ± 0.98	1.84 ± 1.11	1.56 ± 0.69	0.020
TC(mmol/L)	4.21 ± 1.27	4.33 ± 1.15	3.99 ± 1.43	0.063
HDL-C(mmol/L)	1.16 ± 0.28	1.12 ± 0.26	1.23 ± 0.29	0.004
LDL-C(mmol/L)	2.41 ± 0.94	2.37 ± 0.98	2.47 ± 0.87	0.475
LPa(mmol/L)	62.48 ± 84.89	70.69 ± 98.11	47.97 ± 51.49	0.027
FBG(mmol/L)	6.66 ± 2.73	6.91 ± 3.01	6.23 ± 2.10	0.053
BNP(pg/mL)	84.62 ± 144.59	98.19 ± 163.72	60.63 ± 98.84	0.037
hs-CRP(mg/L)	3.00 ± 4.34	2.92 ± 3.77	3.13 ± 5.22	0.730

Continuous variables are presented as mean value ± 1 SD, whereas categorical variables are presented as absolute and relative frequencies

ACS, acute coronary syndrome; STEMI, ST-elevated myocardial infarction; NSTEMI, non ST-elevated myocardial infarction; UAP, unstable angina pectoris; DM, diabetes mellitus; BMI, body mass index; TG, Triglyceride; TC, total cholesterol; LDL-C, low-density lipoprotein-cholesterol; HDL-C, high-density lipoprotein-cholesterol; LPa, lipoprotein a; FBG, fasting blood glucose; BNP, B-natriuretic peptide; hs-CRP, hypersensitive C-reactive protein

### Pro-inflammatory cytokine plasma levels and ceramides of participants

Table 2 presents the plasma levels of 3 pro-inflammatory cytokines and 7 ceramide species in all participants included in the study. Patients with ACS exhibited significantly higher levels of TNF-α and IL-6 compared with those without ACS (*P* < 0.01). Among the analysed ceramides, ceramide (d18:1/16:0) displayed the most substantial elevation in plasma levels in the patients with ACS, with a *P*-value close to 0. The *P*-values for ceramide (d18:1/24:0) and ceramide (d18:1/22:0) were also close to 0, indicating significant elevation. For ceramide (d18:1/18:0) and ceramide (d18:1/20:0), the *P*-values were < 0.05, indicating a significant increase. In contrast, the *P*-values for ceramide (d18:1/14:0) and ceramide (d18:1/24:1) were 0.097 and 0.361, respectively, suggesting no significant elevation. These findings suggest that the plasma levels of ceramide (d18:1/24:0), ceramide (d18:1/22:0), ceramide (d18:1/18:0) and ceramide (d18:1/20:0) were significantly elevated in the patients with ACS, while no significant elevation was observed for ceramide (d18:1/14:0) or ceramide (d18:1/24:1).

**Table 2:** Plasma levels of proinflammatory cytokines and ceramide molecules

Variables	total(n = 216)	ACS(n = 138)	non ACS(n = 78)	*P*
TNF-α(pg/mL)	11.75 ± 8.78	13.04 ± 9.21	9.47 ± 7.47	0.004
IL-6(pg/mL)	7.59 ± 5.36	9.09 ± 5.70	5.93 ± 3.99	0.000
IL-8(pg/mL)	24.08 ± 14.14	24.45 ± 13.65	23.41 ± 15.04	0.604
Cer (d18:1/16:0)(µmol/L)	155.17 ± 48.11	165.00 ± 49.09	137.79 ± 41.20	0.000
Cer (d18:1/18:0)(µmol/L)	44.75 ± 18.85	46.71 ± 18.81	41.28 ± 18.54	0.042
Cer (d18:1/24:0)(µmol/L)	1835.10 ± 6761.38	1933.30 ± 710.43	1661.36 ± 559.21	0.004
Cer (d18:1/24:1)(µmol/L)	486.19 ± 193.99	495.28 ± 180.50	470.10 ± 216.09	0.361
Cer (d18:1/14:0)(µmol/L)	2.68 ± 1.36	2.80 ± 1.43	2.48 ± 1.21	0.097
Cer (d18:1/20:0)(µmol/L)	54.10 ± 20.53	56.27 ± 21.37	50.25 ± 18.45	0.038
Cer (d18:1/22:0)(µmol/L)	421.32 ± 159.87	442.57 ± 174.76	383.72 ± 121.56	0.009

Continuous variables are presented as mean value ± 1 SD.

TNF-α, tumor necrosis α; IL-6,interleukin-6;IL-8,interleukin-8;Cer, ceramide

### The association between ceramide and inflammatory factors

Table 3 presents the associations between plasma ceramides and inflammatory factors. The results indicate no significant associations between ceramides and pro-inflammatory cytokines TNF-α, IL-6 or IL-8. However, a mild association was observed between hs-CRP and ceramides d18:1/18:0 and d18:1/20:0, with *P*-values of < 0.5.

**Table 3:** The pearson correlation coefficients between ceramides and inflammatory factors for the whole study population (n = 216)

	TNF-α		IL6		IL8		hs-CRP	
	r	*P*	r	*P*	r	*P*	r	*P*
Cer (d18:1/16:0)	-0.081	0.236	0.027	0.696	-0.030	0.664	0.127	0.063
Cer (d18:1/18:0)	-0.035	0.606	-0.043	0.533	-0.098	0.152	0.138	0.043
Cer (d18:1/24:0)	-0.124	0.069	-0.041	0.544	-0.046	0.497	0.099	0.148
Cer (d18:1/24:1)	-0.070	0.308	-0.084	0.220	-0.066	0.332	0.040	0.186
Cer (d18:1/14:0)	-0.131	0.055	-0.077	0.258	-0.019	0.786	0.087	0.205
Cer (d18:1/20:0)	-0.094	0.171	-0.130	0.057	-0.085	0.216	0.139	0.042
Cer (d18:1/22:0)	-0.136	0.045	-0.099	0.149	-0.050	0.465	0.127	0.063

TNF-α, tumor necrosis α; IL-6, interleukin-6; IL-8, interleukin-8; hs-CRP, hypersensitive C-reactive protein; Cer, ceramide

### Univariable and multivariable logistic regression results: the clinical acute coronary syndrome predictors

Table 4 presents the results of the univariate logistic and multivariate regression analyses for clinical predictors of ACS. Among the traditional risk factors, age (OR = 0.981, 95% CI: 1.580–4.990, *P* < 0.001), male (OR = 2.808, 95% CI: 1.272–4.767, *P* < 0.001), DM history (OR = 2.462, 95% CI: 1.272–4.767, *P* < 0.01) and being a current smoker (OR = 3.961, 95% CI: 1.999–7.848, *P* < 0.001) were significant predictors of ACS (*P* < 0.05). Additionally, most of the lipid profiles, including TGs (OR = 1.405, 95% CI: 1.008–1.960, *P* < 0.05) and HDL-C (OR = 0.227, 95% CI: 0.081–0.636, *P* < 0.01), were significantly associated with ACS (*P* < 0.05). The pro-inflammatory cytokines, TNF-α (OR = 1.063, 95% CI: 1.018–1.109, *P* < 0.01) and IL-6 (OR = 1.157, 95% CI: 1.077–1.243, *P* < 0.001), were also significant predictors of ACS (*P* < 0.01). Moreover, several ceramides, including ceramide (d18:1/16:0) (OR = 1.016, 95% CI: 1.008–1.024, *P* < 0.001), ceramide (d18:1/18:0) (OR = 1.017, 95% CI: 1.000–1.034, *P* < 0.05), ceramide (d18:1/24:0) (OR = 1.001, 95% CI: 1.000–1.001, *P* < 0.01), ceramide (d18:1/20:0) (OR = 1.016, 95% CI: 1.001–1.032, *P* < 0.05) and ceramide (d18:1/22:0) (OR = 1.003, 95% CI: 1.001–1.005, *P* < 0.05), were significantly associated with ACS.

**Table 4:** The significant predictors for ACS patients in univariate and multivariate logistic regression

Variables	Univariate	*P* value	Multivariate	*P* value
	OR(95%CI)		OR(95%CI)	
Age	0.981 (1.580–4.990)	0.000		
Male	2.808 (1.272–4.767)	0.000	2.702 (1.290–5.658)	0.008
DM	2.462 (1.272–4.767)	0.008	2.329 (1.077–5.035)	0.032
Hypertention	1.176 (0.668–2.070)	0.574		
Current smoker	3.961 (1.999–7.848)	0.000	2.702 (1.204–6.066)	0.016
Alcohol	2.221 (0.997–4.951)	0.051		
BMI	0.922 (0.862–0.987)	0.02		
TG	1.405 (1.008–1.960)	0.045		
TC	1.240 (0.987–1.557)	0.065		
HDL-c	0.227 (0.081–0.636)	0.005		
LDL-c	0.898 (0.669–1.205)	0.474		
LPa	1.004 (1.000-1.008)	0.065	1.006 (1.000-1.012)	0.04
FBG	1.108 (0.986–1.245)	0.084		
BNP	1.002 (1.000-1.005)	0.083		
hs-CRP	0.989 (0.928–1.053)	0.729		
TNF-α	1.063 (1.018–1.109)	0.005		
IL-6	1.157 (1.077–1.243)	0.000	1.173 (1.082–1.271)	0.000
IL-8	1.005 (0.985–1.026)	0.603		
Cer(d18:1/16:0)	1.016 (1.008–1.024)	0.000	1.018 (1.008–1.028)	0.000
Cer(d18:1/18:0)	1.017 (1.000-1.034)	0.045		
Cer(d18:1/24:0)	1.001 (1.000-1.001)	0.005		
Cer(d18:1/24:1)	1.001 (0.999–1.002)	0.362		
Cer(d18:1/14:0)	1.205 (0.965–1.505)	0.100		
Cer (d18:1/20:0)	1.016 (1.001–1.032)	0.041		
Cer (d18:1/22:0)	1.003 (1.001–1.005)	0.011		

OR,odds ratio; CI, confidence interval; DM, diabetes mellitus; BMI, body mass index; TG, Triglyceride; TC, total cholesterol; LDL-C, low-density lipoprotein-cholesterol; HDL-C, high-density lipoprotein-cholesterol; LPa, lipoprotein a; FBG, fasting blood glucose; BNP, B-natriuretic peptide; hs-CRP, hypersensitive C-reactive protein; TNF-α, tumor necrosis α; IL-6, interleukin-6; IL-8, interleukin-8; Cer, ceramide

The results of the multivariate regression analysis demonstrated that being male (OR = 2.702, 95% CI: 1.290–5.658, *P* < 0.01), having a DM history (OR = 2.329, 95% CI: 1.077–5.035, *P* < 0.05), being a current smoker (OR = 2.702, 95% CI: 1.204–6.066, *P* < 0.05), LPa (OR = 1.006, 95% CI: 1.000–1.012, *P* < 0.05), IL-6 (OR = 1.173, 95% CI: 1.082–1.271, *P* < 0.001) and ceramide (d18:1/16:0) (OR = 1.018, 95% CI: 1.008–1.028, *P* < 0.001) were all significant predictors of ACS.

### Predictive value of the models for acute coronary syndrome

**Fig. 2:**
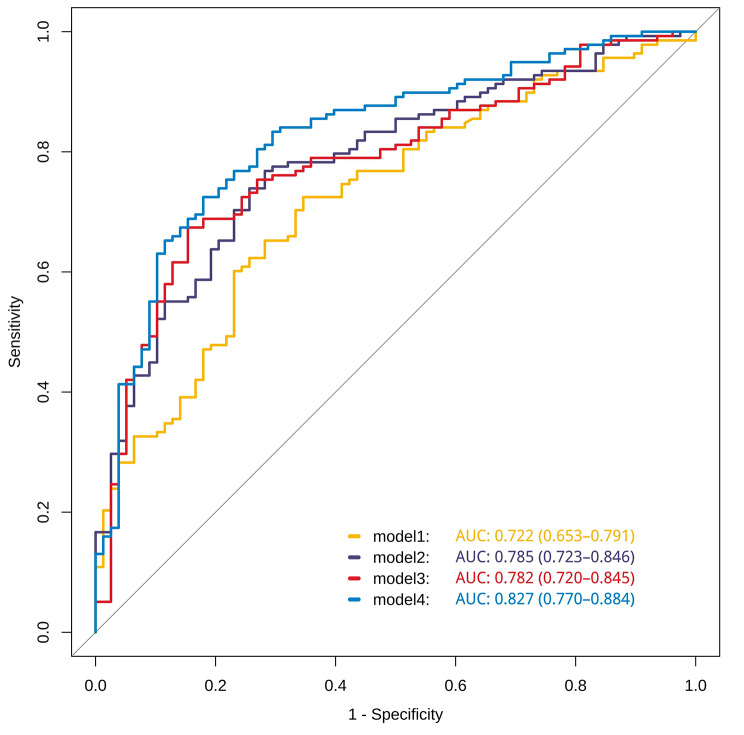
The ROC of the four diagnostic models

Table 5 presents the predictive values of four diagnostic models, which included variables with *P*-values of < 0.05 in the multivariate logistic regression. Model 1 achieved an area under the curve (AUC) of 0.722 for diagnosing ACS. When IL-6 was added to the model, the AUC increased to 0.785. Incorporating traditional risk factors along with ceramide (d18:1/16:0) resulted in an AUC of 0.782. Finally, model 4, which combined traditional risk factors (male gender, history of DM, current smoking status and elevated LPa, IL-6 and ceramide [d18:1/16:0]), demonstrated an AUC of 0.827. The results indicated that the combination of traditional risk factors, IL-6 and ceramide (d18:1/16:0) significantly improved the AUC of model 4 compared with model 1 (0.827 [0.770–0.884] vs. 0.722 [0.653–0.791], *P* < 0.001), model 2 (0.827 [0.770–0.884] vs. 0.785 [0.723–0.846], *P* < 0.05) and model 3 (0.827 [0.770–0.884] vs. 0.782 [0.720–0.845], *P* < 0.05). The ROC curves for the prediction of ACS for the four models are depicted in Fig. 2.

**Table 5:** The predictive value of the four models for ACS patients

Old model	New model	AUC(95%CI)	*P* value -AUC
Model 1	-	0.722 (0.653-0.791)	
Model 1	model 2	0.785 (0.723-0.846)	0.01
Model 1	model 3	0.782 (0.720-0.845)	0.015
Model 1	model 4	0.827 (0.770-0.884)	0.001

*P* values AUC for the difference between old model and new model .AUC,area under the curve

Model 1: included male,DM history,current smoker,LPa;

Model 2:model 1 + IL 6;

Model 3:model 1 + ceramide(d18:1/16:0);

Model 4:model 1 + IL 6 + ceramide (d18:1/16:0)

## Discussion

Due to the multifaceted nature of the underlying aetiology and mechanisms of ACS, relying on a single biomarker for accurate prediction is unlikely. Therefore, the development of a multi-marker model is crucial to enhance the prediction of ACS occurrence. In this study, a proportional increase in the plasma levels of ceramide (d18:1/16:0), ceramide (d18:1/18:0), ceramide (d18:1/20:0), ceramide (d18:1/22:0), ceramide (d18:1/24:0), TNF-α and IL-6 in patients with ACS was observed. Certain ceramide species, specifically, ceramide (d18:1/16:0), ceramide (d18:1/18:0), ceramide (d18:1/20:0), ceramide (d18:1/22:0) and ceramide (d18:1/24:0), along with TNF-α and IL-6, were identified as independent predictors of ACS, even after adjusting for traditional risk factors.

The proposed model, incorporating the traditional risk factors, ceramide (d18:1/16:0) and IL-6, demonstrated an AUC of 0.827. In comparison, the AUCs of the models considering only the traditional risk factors ceramide (d18:1/16:0) or IL-6 individually were 0.782, 0.785 and 0.722, respectively. These findings suggest that combining the assessment of traditional risk factors, including male gender, history of DM, current smoking status and elevated LPa with ceramide (d18:1/16:0) and IL-6, can enhance the predictive accuracy for ACS.

Although our study did not find any significant associations between the ceramide subspecies and proinflammatory cytokines, in-vitro research suggests that ceramide, as a second messenger of sphingolipids, is linked to various cytokines, including TNF-α and IL-6. Ceramides have been shown to stimulate the secretion of IL-6 and CRP, exerting a direct pro-inflammatory effect [24–28]. Some studies have indicated that cytokines such as TNF-α and IL-6 can impact phospholipid metabolism and subsequently influence ceramide production [18]. However, our study did not find a significant association between any of the ceramide molecules and TNF-α or IL-6, while a mild relationship was observed between hs-CRP and ceramide (d18:1/18:0) and ceramide (d18:1/20:0), which is inconsistent with prior studies. Existing studies refer to research that indicated that TNF-α and IL-6 could impact phospholipid metabolism and, subsequently, influence ceramide production; our study, however, did not find a significant association between any of the ceramide molecules. This discrepancy may be attributed to our relatively small sample size and the lack of serial biomarker measurements. Further research is warranted to explore the relationship between proinflammatory cytokines and ceramide molecules in more detail.

Numerous studies have provided compelling evidence of the strong association between inflammation and ACS [29]. Interleukin-6, IL-18 and TNF-α are found in human plaques and may play roles in plaque progression and rupture since they have been associated with ACS. Additionally, they are associated with a higher incidence of acute cardiovascular events in patients with extreme cardiovascular risk [30]. Among the proinflammatory cytokines, IL-6 (primarily produced by T-cells and macrophages) plays a crucial role in destabilising plaques, promoting atheroprogression and stimulating the production of hs-CRP [31, 32]. Moreover, a comprehensive analysis of multiple studies consistently demonstrated that elevated blood levels of IL-6 independently increase the risk of major adverse cardiovascular events, as well as cardiovascular and all-cause mortality in patients with ACS [33]. Consequently, measuring IL-6 levels in the blood holds promise for improving the risk stratification of patients with ACS [34]. In our study, we observed significantly higher plasma levels of IL-6 in patients with ACS; furthermore, we identified an independent association between IL-6 levels and the occurrence of ACS.

Ceramides, which are bioactive lipids with crucial regulatory functions in pro-inflammatory cytokines, have been implicated in various cardiovascular conditions. Elevated serum ceramide concentrations serve as predictors for cardiovascular atherosclerotic disease, stroke, heart failure and atrial fibrillation [35, 36]. Moreover, specific plasma ceramide levels are correlated with heightened cardiovascular mortality in ambulatory patients with chronic heart failure [37]. A previous study developed a model that combined the measurement of ceramides with high-sensitive troponin T for the detection of ACS in patients presenting with chest pain, achieving an impressive AUC of 0.865 [38]. Another study demonstrated that elevated plasma levels of ceramide (d18:1/16:0), ceramide (d18:1/18:0) and ceramide (d18:1/24:1) were independent predictors of a high atherosclerotic burden in patients with STEMI [39]. Furthermore, ceramide molecules, including ceramide (d18:1/16:0), ceramide (d18:1/18:0) and ceramide (d18:1/24:1), as well as their ratios to ceramide (d18:1/24:0), have emerged as promising risk stratifiers in patients with established CAD [14]. Additionally, ceramides have shown potential as plasma biomarkers for the early prediction of restenosis after percutaneous coronary intervention [40]. However, this study indicated that only ceramide (d18:1/16:0) independently predicted the occurrence of ACS.

### Limitations

Several limitations of the current study should be acknowledged. First, the study was conducted at a single centre, limiting the generalisability of its findings to other populations. Second, the sample size was relatively small, warranting the need for larger prospective studies to validate the conclusions. Third, the absence of serial measurements hindered the ability to establish a temporal relationship between the biomarkers and the onset of ACS. Fourth, uric acid has been identified as a significant determinant of many different outcomes, such as all-cause and cardiovascular mortality, as well as cardiovascular events in patients with chronic coronary syndromes and ACS [41]. However, the biomarker of serum uric acid was not included in this study. Additionally, internal and external validation of the diagnostic models was not performed, which is crucial for assessing their robustness. Therefore, further research is warranted to confirm the diagnostic value of these biomarkers and to establish a validated diagnostic model for ACS.

## Conclusion

In conclusion, ceramide (d18:1/16:0) plays an essential role in predicting ACS. In addition, this study’s results support the idea that the simultaneous measurement of traditional risk factors, IL-6 and ceramide (d18:1/16:0) can improve the diagnostic accuracy of ACS. While these findings may not offer novel perspectives for developing new therapeutic approaches, an ACS risk assessment combining IL-6 and ceramide (d18:1/16:0) presents a unique tool for aiding clinical implementation and decision-making in patients suspected of having atherosclerosis. Additionally, ACS risk assessment has the potential to enhance patients’ adherence to medical therapy and lifestyle changes.

## Data Availability

All data generated or analyzed during this study are included in this published article.
